# Immunotherapy of targeting MDSCs in tumor microenvironment

**DOI:** 10.3389/fimmu.2022.990463

**Published:** 2022-09-05

**Authors:** Hongshu Sui, Shengyi Dongye, Xiaocui Liu, Xinghua Xu, Li Wang, Christopher Q. Jin, Minhua Yao, Zhaoqing Gong, Daniel Jiang, Kexin Zhang, Yaling Liu, Hui Liu, Guomin Jiang, Yanping Su

**Affiliations:** ^1^ Department of Histology and Embryolog, School of Clinical and Basic Medical Sciences, Shandong First Medical University & Shandong Academy of Medical Sciences, Jinan, Shandong, China; ^2^ Department of Pathology, The Second Affiliated Hospital of Shandong First Medical University, Taian, Shandong, China; ^3^ Department of Pathology and Forensic Medicine, School of Clinical and Basic Medical Sciences, Shandong First Medical University & Shandong Academy of Medical Sciences, Jinan, Shandong, China; ^4^ Department of Medicine, School of Medicine, University of Louisville, Louisville, KY, United States; ^5^ Tuberculosis Prevention and Control Institute of Kashgar, Kashgar City, Xinjiang Uygur Autonomous Region, China

**Keywords:** tumor, myeloid-derived suppressor cells, tumor microenvironment, immunotherapy, immune checkpoint blockade (ICB)

## Abstract

Myeloid-derived suppressor cells (MDSCs) are a group of heterogeneous cells which are abnormally accumulated during the differentiation of myeloid cells. Immunosuppression is the main functional feature of MDSCs, which inhibit T cell activity in the tumor microenvironment (TME) and promote tumoral immune escape. The main principle for immunotherapy is to modulate, restore, and remodel the plasticity and potential of immune system to have an effective anti-tumor response. In the TME, MDSCs are major obstacles to cancer immunotherapy through reducing the anti-tumor efficacy and making tumor cells more resistant to immunotherapy. Therefore, targeting MDSCs treatment becomes the priority of relevant studies and provides new immunotherapeutic strategy for cancer treatment. In this review, we mainly discuss the functions and mechanisms of MDSCs as well as their functional changes in the TME. Further, we review therapeutic effects of immunotherapy against MDSCs and potential breakthroughs regarding immunotherapy targeting MDSCs and immune checkpoint blockade (ICB) immunotherapy.

## Introduction

It is known that the incidence of cancer is still rising with poor prognosis and high mortality, despite the continuous improvement of treatment modalities (1). At present, the main treatments for cancer include surgery, radiotherapy, chemotherapy. However, those treatments have severe side effects on healthy cells, and have limitations such as drug resistance. Immunotherapy has increasingly attracted wide attention due to its unique advantages, especially its long-lasting therapeutic effects (2, 3). Recently, it was reported that the immune checkpoint blockade (ICB) immunotherapy, such as those targeting CTLA-4, PD-1, and PD-L1. ICB greatly improved treatment efficacy on tumors with high mutation rates, such as non-small cell lung cancer (NSCLC). In the clinical data statistics of NSCLC, it was found that the composite mutation characteristics are closely related to the pro-inflammatory tumor microenvironment (TME). In addition, the checkpoint proteins are the most commonly used biomarkers for NSCLC patients, especially for high expression of PD-L1 on tumor and PD-1 on T cells (4, 5). However, the response rate for ICB immunotherapy is only 15-20% in various types of solid tumors, which is far from clinical request due to complex TME (6).. Therefore, the impact of cancer on the immune system still needs to be explored.

The immune system consists of many negative feedbacks of inhibitory pathways that are found to suppress the development of excessive immune responses to avoid autoimmune reactions (7, 8). In the process of cancer development, there is a balance complex between cancer and the immune system. The occurrence and development of cancer cause immune escape, promoting cancer progression (9). The long-term inflammatory invasion of cancer cells disrupts the balance of the immune system, making immune cells exhausted, finally leading to continuous tumor growth and metastasis. Therefore, researchers are paying more attention to the anti-tumor immune responses on the TME, where there are plenty of cancer suppressor factors such as tumor-related stromal cells, regulatory T cells (Treg) and immunosuppressive myeloid cells (IMCs) (10). The immunosuppressive cytokines in TME produced by tumor cells, MDSCs and CAFs are crucial factors that mediate T-cell and other immune cell dysfunction. TGF-β signaling pathway, for example, plays an important role in tumor promotion (11, 12). A hallmark of cancer development is persistent inflammatory invasion and immune escape, which induce oncogene protein, reduces tumor suppressor genes, and produces IMCs which include TAMs and MDSCs (13, 14). Plenty of IMCs in TME and their strong inhibitory roles on lymphocytes have become the main obstacle to tumor immunotherapy (15). Here we focus on one type of cell of IMCs, MDSCs, which suppress T cell to promote tumor cell proliferation, metastatic growth and immunotherapy resistance (16).

**Figure 1:**
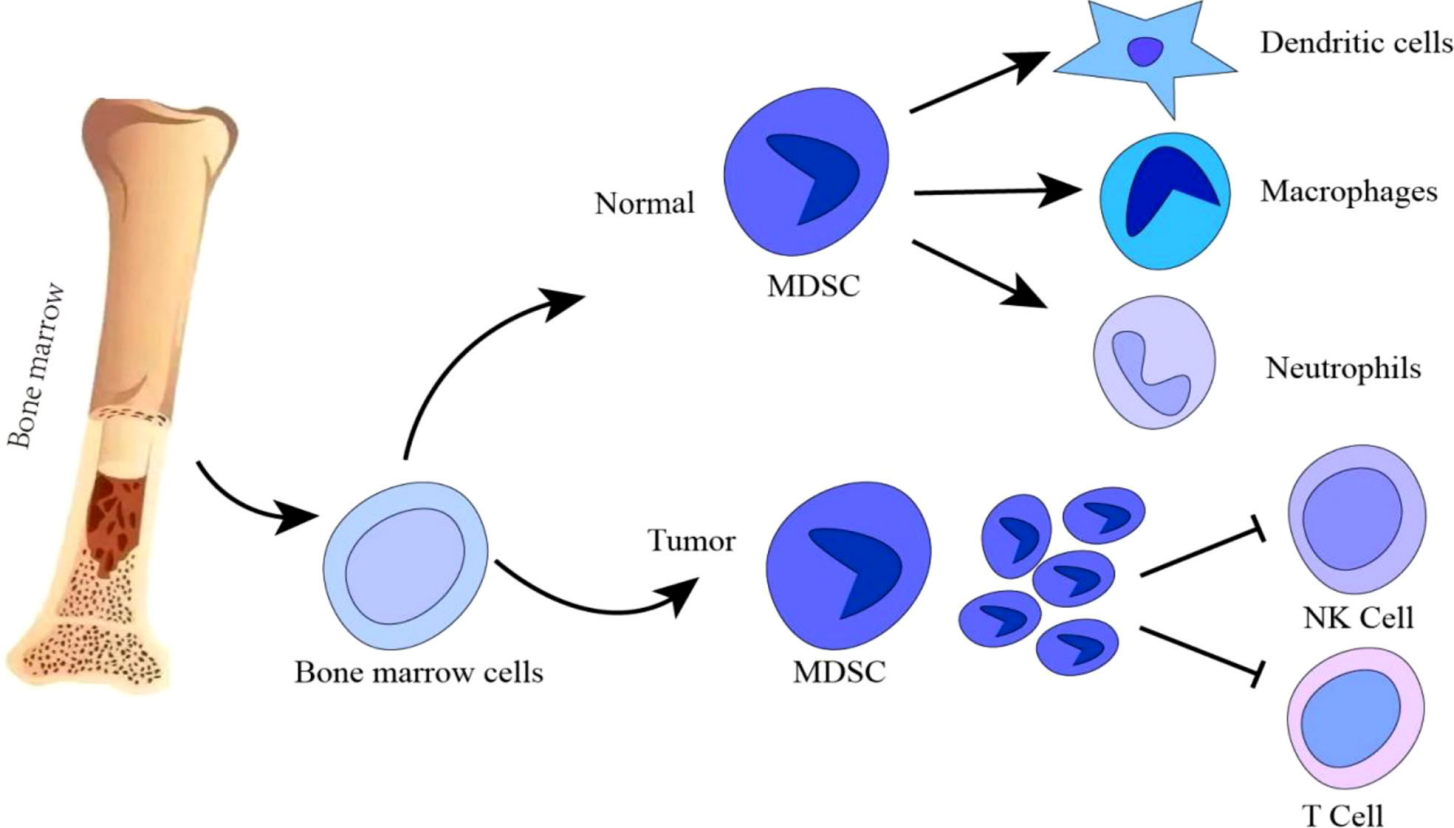
During normal myelogenesis, bone marrow hematopoietic stem cells differentiate into immature cells, which migrate to corresponding peripheral organs and further differentiate into macrophages, dendritic cells or neutrophils. However, in a chronic inflammatory or tumor microenvironment, the differentiation of immature bone marrow cells is blocked, and abnormal accumulation of immature bone marrow cells is induced. Tumor MDSCs are increased to inhibit T/NK cells anti-tumor immune response. Promotion: →; Inhibition: ┤.

MDSCs are a class of highly heterogeneous cells derived from immature myeloid progenitors, consisting of myeloid progenitors and precursors of macrophages, granulocytes and DCs, trigger the abnormal state of hematopoietic stem cells in the differentiation process and display immunosuppressive activity on T cells and NK cells during the progression of cancer (17) (**Figure 1**). MDSCs mainly consist of two cell subsets: granulocytic/polymorphonuclear MDSCs (PMN-MDSCs) and monocytic MDSCs (M-MDSCs) (18). During cancer development, it is stimulated by long-term inflammatory factors and cytokines (e.g., high levels of GM-CSF, VEGF, IL-6, IL-1β, adenosine, HIF1α). This leads to myelogenesis and regulation in the bone marrow, which transforms into pathologically activated cells in tissues; Moderate myelogenesis, changes in cellular metabolism, thereby inhibiting immune function. Immunosuppression roles of MDSCs are mainly regulated through STAT3, STAT1, STAT6, NF-κB, ER stress pathway, Down-regulation of cAMP, COX2, PTGES, CEBPβ, IRF8, RB1 and lipid oxidation (19, 20). MDSCs participate in promoting tumor progression by enhancing tumor angiogenesis and invasion, and facilitating pre-metastatic niche formation (21, 22). Thus, MDSCs play an important role in determining the therapeutic effect of immunotherapy. In this review, we address the regulatory mechanisms of MDSCs as well as their functional changes in the TME, and summarize combined therapeutic effect of targeting tumor MDSCs and ICB.

## Characteristics and functioning mechanisms of MDSC in the TME

**Figure 2:**
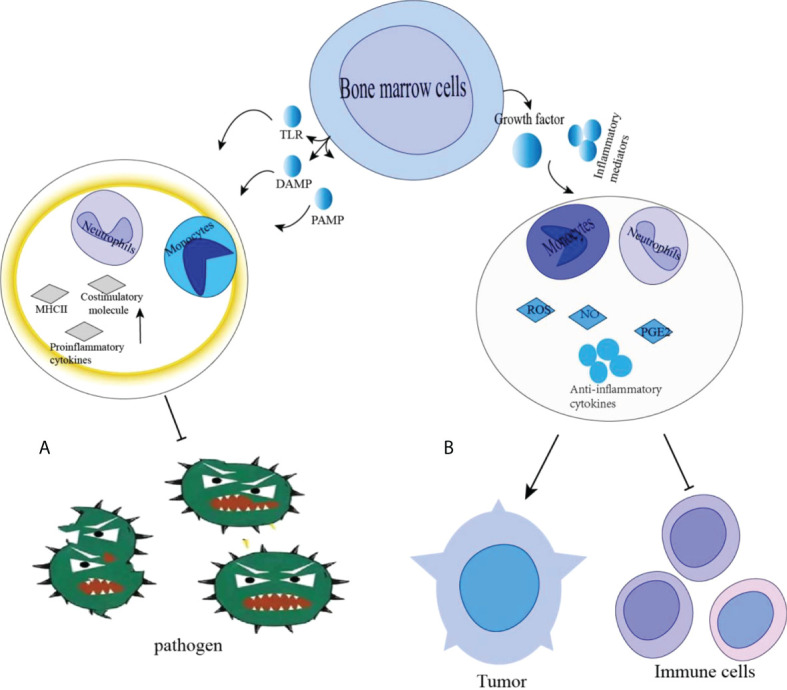
**(A)** Classical activation of bone marrow cells occurs in response to pathogen signals, mainly in the form of Toll-like receptor (TLR) ligands, various damage-related molecular pattern (DAMP) and pathogen-associated molecular pattern (PAMP) molecules. This results in rapid activation of monocytes and neutrophils in the bone marrow, a significant increase in phagocytosis, respiratory bursts, production of pro-inflammatory cytokines and upregulation of major histocompatibility complex (MHC) class II and costimulatory molecules. **(B)** In chronic infections and cancer, Immature cell differentiation is blocked. They exhibit relatively weak phagocytic activity, increased levels of reactive oxygen species (ROS) and nitric oxide (NO) production, arginase and PGE2, promoting tumor growth. Promotion: →, Inhibition: ┤.

During normal myelopoiesis, the major myeloid populations include granulocytes (with the most representative being neutrophils), monocytes, terminally differentiated macrophages (MΦ), and DCs. Those mature myeloid cells are one of the main protective mechanisms against pathogens (23–25) (**Figure 1**). For instance, classical activation of myeloid cells occurs in response to antipathogenic signals, mainly in the form of toll-like receptor (TLR) ligands, various damage-associated molecular pattern (DAMP) molecules, and pathogen-associated molecular pattern (PAMP) molecules (26). The activation leads to the production of monocytes and neutrophils in the bone marrow, markedly increase phagocytosis, a respiratory burst, the production of pro-inflammatory cytokines and the upregulation of both the major histocompatibility complex (MHC) class II and co-stimulatory molecules. This process usually lasts for only a short time and the reaction disappears with the threat (i.e., pathogen) lost. MDSC formation is different from normal myelogenesis, which is a pathological activation state resulting from continuous stimulation of tumor or chronic inflammation on bone marrow compartment, leading to high levels of ROS, MPO, NO, and most anti-inflammatory cytokines, which prevent the generation of mature bone marrow cells (27, 28). C/EBPα and C/EBPβ play the important role in the formation of bone marrow and affect the formation and development of MDSC. C/EBPα makes the granulocyte lineage specific transition from CMP to granulocyte-monocyte precursors (GMPs), GMP. After the GMP phase, C/EBPα is dispensable, while C/EBPβ plays a major role. C/EBP family members have been found to control the expansion and functional properties of MDSC, and may regulate MDSC differentiation into neutrophils and macrophage. Under pathological conditions, C/EBPα may be down-regulated in MDSCs, but C/EBPβ, C/EBP-δ may be strongly up-regulated (29). MDSC-like cells are continuously activated through signal transduction and activator of transcription 3 (STAT3) or NF-κB pathway (20).However, in some chronic infections and cancers, the signals that activate myeloid cells are quite different (24, 30). These signals are relatively weak and long-lasting in the present of growth factors and inflammatory mediators. Both neutrophils and monocytes under these conditions exhibit immature phenotype and morphology, relatively weak phagocytic activity, increased levels of reactive oxygen species (ROS), nitric oxide (NO) production, Arginase and Prostaglandin E2 (PGE2) (23, 26, 30, 31). Those myeloid cells in the pathological state suppress T cell anti-tumor immune response, are now referred to as MDSCs (32, 33) (**Figure 2**).

MDSCs are the main suppressive immune cells with the ability to suppress the adaptive and innate immune responses (34–36). However, the functional mechanisms of MDSCs in immune suppression have been completely uncovered. They can be further divided into two subtypes: PMN-MDSCs and M-MDSCs) (37). PMN-MDSCs are phenotypically and morphologically similar to neutrophils, whereas M-MDSCs are similar to monocytes (38). MDSCs in mice have two distinct subtypes due to different markers on them in mice and humans; In mice, MDSCs are characterized by co-expression of CD11b of α-M integrin, which is considered to be a marker of ubiquitination, and bone marrow differentiation antigen Gr-1 which is a glycosylphosphatidylinositol junction protein, is composed of Ly6C and Ly6G subunits, resulting in two subtypes: CD11b^+^Ly-6G^+^Ly-6C^high^andCD11b^+^Ly-6G^+^Ly-6C^low^ (39). Since the CD11b^+^ Ly-6G^+^ Ly-6 C^high^ subtype is morphologically similar to monocytes, it is termed M-MDSCs, and the CD11b^+^Ly-6G^+^Ly-6C^low^ subtype displays a granulocyte-like morphology and is termed granulocytic MDSCs (PMN-MDSCs or G-MDSCs) (34). In human, M-MDSC expression is characterized by CD11b, CD14, HLA-DR and CD15 (40). M-MDSCs can be defined as CD11b+CD15-CD14+HLA-DR-/low MDSCs, while PMN-MDSCs are usually defined as CD11b+CD14-CD15+ (or CD66b+) MDSCs (20, 33). Recently, third subgroup of MDSCs in humans are found to be called early MDSCs, which lacks expression of mature blood cell markers (including CD3, CD14, CD15, CD19 and CD56), thus it is called Lin-HLA-DR-CD33+ (41). consisting mainly of cells with colony-forming potential and other myeloid precursor cells (33, 42).

In the TME, MDSCs are accumulated to suppress immune function and promote tumor growth through inducing some tumor-derived factors, cytokines and/or chemokines such as interleukin (IL)-6, interferon (IFN)-γ, IL-1β, granulocyte-macrophage colony-stimulating factor (GM-CSF), tumor necrosis factor (TNF)-α and vascular endothelial growth factor (43, 44). In Esophageal Squamous Cell Carcinoma (ESCC), the increase of MDSCs is upregulated through IL-6 or other signaling pathways mediated by aldehyde dehydrogenase (6, 45). The continuous recruitment of MDSCs to tumors is also mediated by interactions between chemokines and chemokine receptors, particularly the interaction between the CC chemokine receptor 5 (CCR5) and its ligand to exaggerate ESCC progression. In melanoma, tumor-infiltrating CCR5^+^ MDSCs was found to show elevated expression levels of immunosuppressive markers such as PD-L1, Arg1, ROS and NO, exerting stronger immunosuppressive activity compared with its CCR5^-^ counterparts (12). In breast cancer, BCC-Ex induced the increase of myeloid cells by activating the STAT3 signaling pathway, promoting the expansion of MDSCs (27, 46). In the TME, the immunosuppressive function of MDSCs is also mediated through endoplasmic reticulum stress and inhibitor-related enzymes, which include cyclooxygenase 2(COX2), NADPH oxidase 2 (NOX2), Indoleamine 2, 3-dioxygenase (IDO) and arginase 1 (ARG-1) which induces nitrogen Nitric oxide synthase (iNOS or NOS2) (47). MDSCs also regulate the functional activity of other immune cells, such as macrophages, NK cells, Treg, and B cells (48, 49). In a mouse non-T-cell inflammatory oral cancer model (MOC2), peripheral CXCR2^+^ PMN-MDSCs are pathologically accumulated to enter tumors, inhibiting the functions of NK cells (50, 51). MDSCs also interact with other IMCs such as TAMs to promote their immunosuppressive activities (37, 52) **Tables 1**, **2**.

**Table 1:** Roles of cytokine and chemokines on tumor MDSCs.

Tumor	Cytokines/Signaling pathways	Functions	Ref
**BC** **BC** **ESCC** **Melanoma**	BCC-Ex/CXCR4 BCC-Ex/STAT3 IL-6 CCR5	To increase MDSC and inhibit T cells To promote MDSC expansion To regulate the activation of MDSC To inhibit immune activity	(27) (46) (44) (12)

BC, Breast cancer; ESCC, Esophageal squamous cell carcinoma.

**Table 2:** Function of target protein on tumor MDSCs.

Tumor	Target	Function	Ref
**RCC** **NSCLC** **MOC2** **HNSCC** **Neuroblastoma** **Melanoma**	IL-1β/PD-1 Gr-1/ATRA CXCR2 CXCR1/2 NKG2D LXR/Apoe	To reduce the PMN-MDSCs To block G-MDSC To regulate NK cell function To modulate immune activity To improve the ability of T cells To change the number of MDSCs	(56) (57) (50, 51) (59) (60) (61)

RCC, Renal cell Carcinoma; NSCLC, Non-small cell lung cancer; MOC2, Oral cancer model; HNSCC, Head neck Squamous Cell Carcinoma.

MDSCs exert its immunosuppressive function through the induction of different cytokines or chemokines. Porta et al. found that inhibition of PGE2/P50/NO axis could prevent the inhibitory function of MDSC and restore the functional effect of anticancer immunotherapy. IFNγ treatment block M-MDSC to produce the tumor-promoting molecule nitric oxide (NO) and PGE2, which promote nuclear accumulation of P50 NF-κB in M-MDSCs, indicating that IFNγ treatment may reverse NO-mediated immunosuppression of MDSCs through PGE2/NO/P50 pathways (53). It has been found in prostate cancer studies that IL-23 produced by MDSCs can promote the development of prostate cancer through activating androgen receptor pathways in castration-resistant prostate cancer (CRPC), promoting cell survival and proliferation in androgen deficiency. Therefore, IL-23 blockage can resist MDSC-mediated castration resistance and produce synergistic effects (54). The expansion of MDSCs is induced in melanoma through NLRP3/IL-1 signaling, suggesting that NLRP3 inhibitors may restore T cell function through reducing the numbers of tumor MDSCs, and the combined treatment with NLRP3 inhibitor and anti-PD-1 is more effective (55). Therefore, Immunotherapy targeting related cytokines or chemokines reverses the suppressive roles of MDSCs on T cells.

## Therapeutic effects of MDSCS on immunotherapy

In tumor treatment, the durable effects of surgery, radiotherapy, and chemotherapy are relatively low, and immunotherapy has a relatively higher and long-lasting treatment outcome. However, Our data have demonstrated that drug resistance is common in most current immunotherapies due to the immunosuppressive cells in the TME, where MDSC-mediated immunosuppression represents a potential mechanism of resistance to immunotherapy (36). Targeting MDSCs may be an important strategy to overcome immunotherapeutic resistance. In renal cell carcinoma (RCCs), it was found that the recruitment of MDSCs into the tumors induced by a tumor-promoting factor, IL-1β led to immune-suppression on cancer cells. After combined treatment with both anti-IL-1β and anti-PD-L1 antibodies, the number of PD-L1+PMN-MDSCs was reduced in the tumors, blocking tumor progression (56). In an LKB1-deficient NSCLC mouse model, ELR+CXC chemokine promoted NSCLC development and increased levels of ELR+CXC chemokines were positively correlated with the abundance of G-MDSCs in the TME. The depletion of G-MDSCs by anti-Gr-1 antibodies or functional blockade of G-MDSCs by ATRA reverses immunosuppression and made LKB1-deficient tumors sensitive to anti-PD-1 treatment (27, 57).

In head and neck squamous cell carcinoma (HNSCC), the infiltration numbers of CXCR1/2^+^CD15^+^ PMN-MDSCs and CD14^+^ M-MDSCs with immunosuppressive function are significantly increased. Researchers inhibit the infiltration of CXCR2^+^ PMN-MDSCs into MOC2 tumors through dual inhibitors of CXCR1/2 and SX-682 to enhance the CTL infiltration and increase the therapeutic effects of adoptive transfer of NK cells (12). In neuroblastoma patients, researchers have developed genetically modified NK cells with chimeric receptors NKG2D, which is a cytotoxic receptor activated by non-classical MHC molecules expressed during stressful events such as DNA damage, hypoxia or viral infection. NKG2D is fused to the ζ chain of the cytotoxic T cell receptor (NKG2D.ζ) to become NKG2D.ζ-NK cells (58). Targeting MDSCs with NKG2D.ζ-NK cells inhibits the function of MDSCs and improves the anti-tumor role of tumor-directed chimeric antigen receptor (CAR)-modified T cells (CAR-T) in a xenograft tumor model (59). The liver X nuclear receptor (LXR)/apolipoprotein E (ApoE) axis has been implicated in enhancing anti-tumor activity. LXRβ and LXRα are two members of the nuclear hormone receptor transcription factor family that drive the transcriptional activation of ApoE and other genes involved in cholesterol, fatty acid, and glucose metabolism. Studies have confirmed that LXR and its transcriptional target ApoE can reduce the abundance of MDSCs to inhibit melanoma development (60). Therapeutic LXR agonists reduced the abundance of MDSCs in a mouse tumor model (61). Therefore, Targeting MDSCs treatment may improve the immunotherapy results on the TME.

In the TME, some immunoglobulins are co-expressed in immunosuppressive cells, influencing their immunosuppressive roles (62). The tandem action of CD39 and CD73 ectonucleotidases expressed on MDSCs can convert ATP to adenosine on Tregs which is thought to be important mediators of immunosuppression in the TME (63, 64). In NSCLC and melanoma, it was found that high expression levels of CD39 and CD73 on tumor MDSCs are positively correlated with tumor progression (65, 66). TGF-β was found to trigger the phosphorylation of mammalian targets of rapamycin, which subsequently activated hypoxia-inducible factor-1α (HIF-1α) that induced CD39/CD73 expression on MDSC to inhibit T cell function. Therefore, CD39/CD73 on MDSCs may be the novel therapeutic target for tumor treatment (67, 68). Their expression levels on MDSCs are reduced to slow down the tumor progression (69). In the TME, the myeloid cell receptor tyrosine kinases (RTKs) (i.e., TYRO3, AXL, and MERTK) and their ligands (i.e., Gas 6 and protein S) suppress immune responses. In tumor-bearing mice, the expressions of TYRO3, AXL, and MERTK, and their ligands increased >20 folds in M-MDSCs and >15 folds in PMN-MDSCs. *Mertk* ^-/-^, *Axl* ^-/-^ and *Tyro3* ^-/-^ tumor models were revealed to reduce RTK enzymatic activity, exhibit defective MDSC roles, and display poor tumor migration capacity to tumor-draining lymph nodes (TDLN) (70). The inhibition of TYRO3, AXL, and MERTK reduced the immune suppression function of MDSCs in a STAT3-dependent manner, increased CD8^+^ T cell infiltration and enhanced treatment efficacy of anti-PD-1 treatment on melanoma (71, 72). These findings suggest that TYRO3, AXL and MERTK, are immunosuppressive and innate immune checkpoint protein, whose inhibitors may improve the TME through downregulating the roles of MDSCs.

CD33 is composed of type 1 membrane proteins and is a transmembrane sialic acid-binding immunoglobulin-like lectin (SIGLEC) possessing two immunoglobin domains that bind to sialic acid and intracellular immunoreceptor tyrosine inhibitory motif (ITIM) (73). CD33 is pathologically overexpressed on MDSCs in blood and tumor tissues from cancer patients to promote tumor growth. Therefore, targeting CD33^+^ MDSCs can effectively reduce the immunosuppression of MDSCs, slowing down tumor growth (74).

## Synergistic roles of ICB and targeting MDSCs

ICB and CAR-T therapies have provided further advantages for cancer immunotherapy (75). Immune checkpoint inhibitors target immune checkpoint molecules, primarily PD-1, PD-L1, and CTLA-4 to restore anti-tumor immune function. Even though some of checkpoint proteins (such as PD-L1) are expressed on the surfaces of both tumor and MDSCs and a few cancer patients show good long-lasting clinical effects after ICB treatment, immunotherapeutic resistance develops in the late stage of most solid tumors.

MDSCs in the TME induced by chemokines or cytokines become major obstacle to compromise the effect of ICB (76, 77). Targeting MDSCs may be a potential breakthrough for ICB (78). It is reported that single immunotherapy treatment on the malignant cholangiocarcinoma (CCA) is not effective due to complex TME. In a mouse model of CAA, PD-L1 is found to be mainly derived from TAMs and MDSCs, promoting tumor progression (79). The response rate for anti-PD-1 treatment is less than 10%. One of the reasons is that the compensatory G-MDSCs mediate immune evasion by impairing the T cell response, and the blockage of TAM alone fails to slow down tumor progression (80). Indeed, the survival time in mice with CCA was longer after combined treatment of G-MDSCs-specific antibodies (i.e., anti-Ly6G antibody, anti-PD-1 antibody and anti-CSF1R antibody), compared with single-antibody treatment (81). In an advanced gastric cancer mouse model, it was found that anti-PD-1 treatment alone was ineffective, since strong infiltration of PMN-MDSCs into tissues inhibited the immune function of CD8^+^ T cells by increasing the expression of some chemokines or cytokines (i.e., ROS, NO, arginase-1, PGE-2) and interacting with PD-L1/PD-1. Therefore, combination therapy of both blocking PD-1 and targeting MDSCs may overcome the drug resistance in the single immune checkpoint inhibitor treatment (82). In the TME, hypoxia attracts immunosuppressive cells such as MDSCs and TAMs, which are important components of TME, upregulated the expressions levels of both immune checkpoint receptors (such as PD-1 and CTLA-4) and their ligands (such as PD-L1, PD-L2, CD80, and CD86), and induced rapid and selective upregulation of PD-L1 on MDSCs, which is mainly dependent on hypoxia-inducible factor 1α (HIF-1α) rather than HIF-2α. The blockage of PD-L1 under hypoxia reverses the immunosuppressive roles of MDSCs on T cell activation, accompanied by down-regulation of IL-6 and IL-10 in MDSCs. Thus, co-blocking of PD-L1 and HIF-1α reduces the immunosuppressive activity of both MDSCs and TAMs to inhibit tumor development (83). In HNSCC, it was found that the efficacy of anti-CTLA-4 treatment was not significant, mainly due to the recruitment and accumulation of MDSCs. The combined treatment of both G-MDSCs depletion and CTLA-4 inhibitor can achieve an excellent anti-tumor treatment (50). In triple-negative breast cancer (TNBC), aberrant SMAD3 activation promotes metastasis of TNBC through the recruitment of MDSCs. SMAD3 is identified as a non-histone substrate of lysine acetyltransferase 6A (KAT6A). Targeting KAT6A in combination with anti-PD-L1 therapy in TNBC-bearing xenografts models reduced MDSC recruitment, significantly alleviated metastasis potential and increased overall survival (84). For advanced prostate cancer (PCa), the majority of patients are resistant to ICB, due to the accumulation of MDSCs. ICB synergizes with targeting MDSCs therapy with multikinase inhibitors (such as cabozantinib and BEZ235) exhibited stronger anti-tumor activity (85). The inhibition of CXCR4 can promote T-cell infiltration through diminishing the immunosuppressive roles of MDSCs in pancreatic ductal adenocarcinoma (PDAC) (86, 87). Indeed, a clinical study using the combined treatment, including CXCR4 antagonist BL-8040 (motixafortide), Pembrolizumab and chemotherapy has been displayed to increase CD8^+^ effector T cell infiltration and decrease MDSC infiltration, leading to tumor suppression (88). Therefore, the combination immunotherapy of ICB and targeting MDSCs may provide some evidence for their clinical efficacy **Table 3**.

**Table 3:** Combination immunotherapy of targeting MDSCs and ICB .

Tumor	ICB Target	Synergistic roles	Ref
**CCA** **GC** **Cancer** **HNSCC** **TNBC** **PCa** **PDAC**	ly6G/PD-1/CSF1R PD-1 PD-L1/HIF-1α CTLA-4 KAT6A/PD-1 BEZ235/mCRPC CXCR4/PD-1	To inhibition G-MDSC To inhibition resistance of ICI To inhibit tumor development To enhance sensitivity of CTLA-4 inhibition To reduce the recruitment of MDSCs To antitumor activity To reduce MDSC	(78) (80) (83) (84) (84) (85) (86, 87)

CCA, Cholangiocarcinoma; GC, Gastric carcinoma; HNSCC, Headneck squam ous Cell Carcinoma; TNBC, Triple-Negative Breast Cancer; PCa, prostate cancer; PDAC, Pancreatic ductal adenocarcinoma.

Recent studies found that serine/threonine kinase PIM1 was upregulated in MDSCs in melanoma, which was closely associated with increased FAO and PPARγ-driven lipid metabolism in MDSCs. the increased expression of PIM is negatively related to the therapeutic effect of ICB (89, 90). The inhibition of PIM1 on MDSCs led to the reduced number of MDSCs in the TME, restoring the anti-tumor function of cytotoxic T cells, improving the efficacy of PD-L1 blockade, and overcoming the resistance in ICB-resistant patients (91). In PDAC, CD200 (OX-2; OX-90), a regulator of myeloid cell activity, whose expression is upregulated (92). In preclinical studies, the expression levels of CD200 were elevated in MDSCs in PDAC. The blockade of anti-CD200 antibody reduced the number of intra-tumoral MDSCs, restricting PDAC tumor growth and significantly enhancing the anti-tumor efficacy of ICB and anti-PD-1 antibody (92, 93). Therefore, in the TME, targeting both MDSCs and their surface proteins may reverse the immunotherapy resistance to tumor, providing the theoretical basis and potential breakthrough for clinical treatment of cancer using immunotherapies.

## Potential of immunometabolic therapy in MDSC

During tumor development, competent metabolic programs promote the proliferation and migration of tumor cells and enhance the immunosuppressive tumor microenvironment (TME) (94, 95). In addition, the exaggerated metabolic activity allows cancer cells to hijack essential nutrients and outcompete neighboring invasive immune cells, thereby weakening anti-tumor immunity (96). As the main heterogeneous population of immunosuppressive cells, MDSCs can be regulated by various mechanisms to affect tumor development, and can also change the metabolic environment of its surrounding environment to exert immunosuppressive function (97, 98). It has been found that the combination of bone marrow (BM) precursors with GM-CSF and IL-6 *in vitro* has been found to activate L-arginine metabolic enzymes responsible for the immunosuppressive potential of MDSC. The inhibition of L-arginine metabolism enzymes in MSC-1 cells, one cell line derived from primary MDSC, was found to reduce AMPK activity in MSC-1 cells. Subsequently, the inhibition of AMPK activity by specific inhibitor compound C (COMP-C) resulted in the inhibition of L-arginine metabolic enzyme activity to eliminate the immunosuppressive activity of MDSCs (99, 100). The glycolytic metabolite phosphoenolpyruvate (PEP), an important antioxidant, can prevent excessive ROS production, thus reducing the accumulation of MDSCs and inhibiting their immunosuppressive roles. These findings suggest that glycolytic metabolites play an important role in regulating MDSC and may be a potential target (101). In addition, fatty acid metabolism is also proved to be an important part of the development and functional role of MDSC (100, 101). Polyunsaturated fatty acids (PUFA) promote the accumulation of MDSC and enhances the immunosuppressive function of MDSC on T cells through activating the JAK/STAT3 pathway. JAK inhibitor JSI-124 almost completely prevented the effect of PUFA on MDSCs, indicating that fatty acid metabolism may play an important role in the function of MDSC (102, 103). Similar increases in fatty acid uptake and FAo-related enzyme expression are also observed in MDSCs from blood and tumors in Lewis lung cancer (LLC) and McA-38 colorectal adenocarcinoma mouse models. It was also found that MDSCs promoted the increased fatty acid uptake and activated fatty acid oxidation (FAO) (104). The inhibition of FAO alone delays tumor growth and plays an anti-tumor role. FAO inhibition combined with low-dose chemotherapy can completely inhibit the immunosuppressive effect of MDSC, providing better treatment for anti-tumor therapy (105). Therefore, immune-metabolic therapy is now emerging as a major breakthrough direction in the study of MDSCs, which will provide a new strategy for anti-tumor therapy.

## Conclusion and prospects

The review focuses on immunotherapies against MDSCs and their therapeutic efficacy. In the TME, MDSCs have become the main obstacle to immunotherapy, since they lead to immunotherapeutic resistance. The accumulation of MDSCs can be reduced after targeting chemokines or inflammatory factors, improving the TME and suppressing tumor growth. Moreover, combination treatment with both ICB and targeting MDSCs can effectively inhibit tumor development and progression. These data suggest the effectiveness of targeting MDSCs in TME. Many scientists have conducted in-depth explorations of the immunoglobulins expressed on MDSCs to reveal the regulatory mechanism of these immunoglobulins on the function of MDSCs. However, the functional mechanisms of many immunoglobulins expressed on MDSCs need to be investigated further. In conclusion, immunotherapies targeting MDSCs have significantly increased efficacy and can suppress tumor activity, showing a strong potential to be a new therapeutic strategy for the immunotherapeutic treatment of cancer.

## Author contributions

HS, SD, and XL wrote, reviewed, and revised manuscript, figures and tables. XX and LW prepared the figures and tables. MY, ZG, and YL reviewed and revised the manuscript. HL, SP, CJ, DJ, and GJ revised manuscript, figures and tables. All authors contributed to the article and approved the submitted version.

## Funding

This work was supported by Shandong Medical and Health Science and Technology Development Project (No. 202001010899), Doctor startup fund of Shandong First Medical University (No. 001003053) and Shandong Province graduate education quality curriculum construction project (SDYKC21148, SDYKC18095).

## Acknowledgments

We thank Mr. Steven Qu from MIT who helped to revise and polish the article.

## Conflict of interest

The authors declare that the research was conducted in the absence of any commercial or financial relationships that could be construed as a potential conflict of interest.

## Publisher’s note

All claims expressed in this article are solely those of the authors and do not necessarily represent those of their affiliated organizations, or those of the publisher, the editors and the reviewers. Any product that may be evaluated in this article, or claim that may be made by its manufacturer, is not guaranteed or endorsed by the publisher.
